# Improving Healthcare Worker Resilience and Well-Being During COVID-19 Using a Self-Directed E-Learning Intervention

**DOI:** 10.3389/fpsyg.2021.748133

**Published:** 2021-12-02

**Authors:** Frances Kelly, Margot Uys, Dana Bezuidenhout, Sarah L. Mullane, Caitlin Bristol

**Affiliations:** ^1^Foundation for Professional Development, Pretoria, South Africa; ^2^Johnson and Johnson Health and Wellness Solutions Inc., New Brunswick, NJ, United States; ^3^Johnson and Johnson Global Community Impact, London, United Kingdom

**Keywords:** e-learning, healthcare worker, resilience, wellbeing, mental health, COVID-19, continuing professional development

## Abstract

**Introduction:** High rates of burnout, depression, anxiety, and insomnia in healthcare workers responding to the COVID-19 pandemic have been reported globally.

**Methods:** Responding to the crisis, the Foundation for Professional Development (FPD) developed an e-learning course to support healthcare worker well-being and resilience. A self-paced, asynchronous learning model was used as the training intervention. Each module included practical, skill-building activities. An outcome evaluation was conducted to determine if completing the course improved healthcare worker knowledge of and confidence in the learning outcomes of the course, their use of resilience-building behaviours, their resilience, and their well-being. A secondary objective was to explore if there were any associations between behaviours, resilience, and well-being. Participants completed pre- and post-course questionnaires to measure knowledge of and confidence in the learning outcomes, y, frequency of self-reported resilience-building behaviours, and levels of resilience (CD-RISC) and well-being (WHO-5). Results were analysed in STATA using paired *T*-tests, univariate and multivariate linear regression models.

**Results:** Participants (*n* = 474; 77.6% female; 55.7% primary care) exhibited significant increases in knowledge, confidence, resilience-building behaviour, resilience, and well-being scores. Statistically significant improvements in the frequency of resilience-building behaviours led to significant improvements in resilience (0.25 points; 95% CI: 0.06, 0.43) and well-being (0.21 points; 95% CI: 0.05, 0.36). Increasing changes in well-being scores had a positive effect on change in resilience scores (β = 0.20; 95% CI: 0.11, 0.29), and vice versa (β = 0.28; 95% CI: 0.14, 0.41).

**Conclusion:** A healthcare worker e-learning course can build knowledge and skills that may prompt changes in resilience-building behaviours and improvements in well-being and resilience scores. The findings suggest that e-learning courses may improve more than competency-based outcomes alone but further research is warranted to further explore these relationships.

## Introduction

Healthcare workers (HCWs) are at high-risk of experiencing mental health problems. Exacerbating this issue is the additional stress and trauma brought on by the COVID-19 pandemic. Several factors contribute to the additional stress experienced by frontline HCWs during a pandemic, including longer working hours under difficult conditions; fear of being infected; and making critical decisions regarding the life and death of patients (Huang et al., 2020; Lai et al., 2020; Pfefferbaum, 2020; Senni, 2020). Studies conducted across the world have shown high rates of burnout, depression, anxiety, and insomnia in HCWs responding to the virus (Kang et al., 2020; Lai et al., 2020; Robertson et al., 2020; Senni, 2020). A cross-sectional study conducted in Wuhan, China found high levels of psychological distress in response to the trauma experienced by HCWs working in COVID-19 hospitals (Lai et al., 2020). This finding is consistent with parallel studies which found symptoms of post-traumatic stress disorder (PTSD) among HCWs who were involved in the SARS outbreak (Wu et al., 2009; Brooks et al., 2018).

The work-related consequences of high levels of stress, burnout and fatigue in frontline HCWs has been well-researched in studies conducted after similar pandemics. Burnout is a state of mental, emotional, or physical exhaustion resulting from workplace stressors (World Health Organization [WHO], 2019). The consequences of burnout can include increases in absenteeism, increases in clinical errors, reduced productivity, breaches in infection control, and poorer quality of care (Gray et al., 2019; Robertson et al., 2020). It is important to acknowledge that systemic efforts are necessary to protect HCWs and reduce their exposure to work-related stressors. However, the role of a healthcare worker is inherently stressful, and it is not always feasible to completely remove all work-related stressors. Research has shown that resilience can act as a “buffer” during high periods of stress and is a protective factor against post-traumatic stress disorder and burnout (Mealer et al., 2012; McCann et al., 2013; Arrogante and Aparicio-Zaldivar, 2017). In addition to efforts to reduce external stressors, there is an opportunity to improve HCWs’ ability to cope with and manage stress by building their resilience as a complementary approach to the necessary systemic efforts required.

It is of critical importance that the mental health of frontline HCWs be prioritised within the COVID-19 response planning and implementation. Several strategies which can be implemented in resource constrained settings have been documented and include training and education of HCWs in mental health literacy, self-help, and mindfulness skills; provision of locally relevant educational material; and creating awareness of available resources and how to access them (Gray et al., 2019; Robertson et al., 2020).

In direct response to the immediate needs of HCWs, the Foundation for Professional Development (FPD) developed a short online five-module course for frontline HCWs on resilience and well-being during COVID-19 in order to minimise the personal and work-related consequences of being on the frontline during a crisis. While the primary objective was to meet the needs of the community, researchers noted that evidence of the impact of e-learning on behaviour and quality of care is often lacking (Maunder et al., 2010; Cook et al., 2011; Ruggeri and Brayne, 2013; Lawn et al., 2017). According to Ruggeri and Brayne (2013), “Health education almost always has a social element, and it is important to evaluate more than just learning and skills to fully understand the benefits of a program.” Researchers in this field highlight the importance of assessing behaviour change using pre- and post-training measures that are objective and reliable (Wutoh et al., 2004; Cook et al., 2011; Ruggeri and Brayne, 2013; Lawn et al., 2017). One particular framework proposed by Kirkpatrick identified four levels for evaluating training effectiveness, namely (1) participant reaction to the training experience; (2) learning outcomes; (3) participant’s behaviour change; and (4) impact on system or organisation (Kirkpatrick, 1994).

Therefore, the secondary objective of this programme implementation, was to explore whether the benefit of the e-learning course could be observed across several levels of Kirkpatrick’s framework (Kirkpatrick, 1994). An outcome evaluation was designed to measure HCW resilience-building behaviours, resilience and wellbeing, and exploratory analyses conducted to inform future course evaluation efforts.

## Methods

### Course Design, Content, and Delivery

The course content focused on improving HCWs’ knowledge of stress in the workplace, how it has been exacerbated by COVID-19, and improving their ability to manage stress, trauma, and distress using practical strategies. The content was delivered online through narrated presentation slides and additional resources such as journal articles and websites. The training intervention used an asynchronous learning model allowing the HCWs to work through the modules at their own pace when they had time, however, content needed to be completed within a month. The five modules were delivered in brief “knowledge bites” to prevent HCWs being overloaded with information.

**FIGURE 1:**
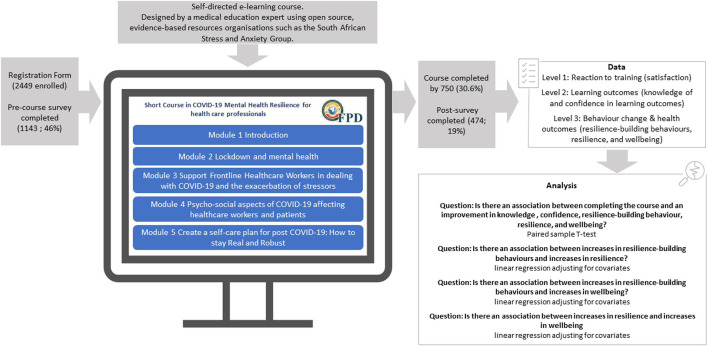
Research design.

The course was designed by a medical doctor with an educational diploma and several years of experience in developing content for training interventions, both online and for contact workshops, and who has developed content for psycho-social rehabilitation, Tuberculosis training for HCWs and other clinical online short courses for General Practitioners. The content of the course was informed by well-known, open source, evidence-based resources that were accessible online (for example resources from the South African Depression and Anxiety Group). Key elements of e-learning interventions, which have been shown to influence learning outcomes include interactivity, skill development, action planning future use of skills, provision of tools for future use, opportunity to practice, repetition, adopting mixed learning approaches, and educational support (Maunder et al., 2010; Lawn et al., 2017; Davies et al., 2018; Regmi and Jones, 2020). As such, the course was designed to be interactive, incorporating quizzes, discussion boards, and practical activities to engage the user and strengthen learning (Figure 1). These activities included self-assessments of burnout and psychological well-being, practicing mindfulness and meditation, and developing a self-care plan. The self-care plan was intended to be used by the HCWs beyond the training and required them to list the self-care practices that they will start engaging in across six categories: Physical (e.g., eat regular and healthy meals, good sleep habits, regular exercise, medical check-ups); Mind/spiritual (e.g., take time for yourself, disconnect from electronic devices, journal, pursue new interests, learn new skills, meditation, access psychotherapy, life coaching, or counselling support); Relationships (e.g., healthy relationships, make time for family/friends, schedule dates with partner/spouse, ask for support from family and friends); Emotions (e.g., engage in positive activities, acknowledge my own accomplishments, express emotions in a healthy way); and Work (e.g., maintain work-life balance, positive relationships with co-workers, time management skills).

### Sample

The sampling method used for this evaluation was non-probability convenience sampling. The sample included all HCWs who had completed the course by December 2020 and who had completed both the pre- and post-training assessments.

At the time of analysis, 2449 HCWs were enrolled in this course and 750 had completed it. Of the 750 completions, 474 HCWs completed both the pre- and post-training assessments and were included in the analysis (Figure 1).

### Data Collection

The evaluation methodology and resultant data collection was based on the first three levels of Kirkpatrick’s framework for evaluating training effectiveness; (1) participant reaction to the training experience; (2) learning outcomes; (3) participant’s behaviour change (Kirkpatrick, 1994). Level 4 data collection and analyses (impact on system or organisation) was out of scope for this evaluation.

The objective of the evaluation was to explore whether the benefit of the e-learning course could be observed across the three levels of Kirkpatrick’s framework. Namely reaction to the training or participant satisfaction (level 1), HCW knowledge of and confidence in the learning outcomes of the course (level 2), and resilience-building behaviours, resilience, and wellbeing (level 3). An exploratory objective was to identify any associations between resilience building behaviours, resilience, and well-being. Data were collected using a satisfaction survey and pre- and post-training assessment that participants completed on the online training portal (Figure 1).

Pre- and post-training knowledge scores were measured using a multiple-choice questionnaire based on the contents of the course (total score of 10). Confidence scores were also measured using a rating scale linked to the learning outcomes of the course (total score of 12). As behaviour could not be directly observed or measured, HCWs were asked to self-report the frequency of engagement in five resilience-building behaviours (total score of 25). These resilience-building behaviours included: using coping mechanisms to deal with stress, engaging in mindfulness and relaxation activities, engaging in self-care behaviours, connecting with a social support network, and discussing the impact of COVID-19 on mental health. Resilience was measured using the validated 10-item Connor-Davidson Resilience Scale (10-item CD-RISC) (Connor and Davidson, 2003). The scale is comprised of 10 items, each rated on a 5-point scale (0–4), with higher scores reflecting greater resilience (total score of 40). The CD-RISC has been validated in the South African context (Jorgensen and Seedat, 2008), specifically in populations that include HCWs (Rogers, 2016a). Well-being was measured using the World Health Organisation-5 well-being index (WHO-5) (World Health Organization [WHO], 1998). The measure comprises of 5 items, each rated on a 6-point scale (0–5), with higher scores suggesting better well-being (total score of 25). The WHO-5 has been used extensively worldwide, including in South Africa (Soares et al., 2009). Demographic data were collected from participants’ registration forms.

### Data Analysis

Mean pre- and post-training scores were calculated and statistically compared using paired sample *t*-test. Crude linear regressions were used to estimate associations between the change (from pre- to post-training) in well-being and resilience and the change in knowledge, confidence, resilience-building behaviours, or socio-demographics individually. Pre-training well-being and resilience scores were included regardless of the *P*-value to account for varying baseline levels. Factors with a *P*-value ≤ 0.05 were included together in a multivariable linear regression model to adjust for potential confounding variables. All model coefficients were interpreted as the mean change of well-being or resilience scores for every one-point increase in the change of each factor of interest, adjusting for other covariates in the model such as socio-demographics, baseline scores, and resilience-building behaviours. Descriptive and multivariable analyses were performed with STATA 13.1 software (StataCorp, College Station, TX, United States).

## Results

### Participant Characteristics

Of the 750 HCWs who had completed the course as of December 2020, 474 completed both the pre- and post-training assessments and were included in the analysis. Most participants were female (77.6%), from South Africa (96.4%), working with outpatients (37.1%), in an urban setting (64.5%), in the public sector (51.1%) (Table 1). A variety of occupations participated and completed the course with the top three being: (1) medical practitioners (16.1%), (2) Physio, Chiro, Dietician, Biokineticist, Podiatry (14.0%), and (3) Registered counsellor, psychologist, social worker (14.0%) (Table 1).

**TABLE 1:** Participant characteristics.

**Characteristic**	**Number (%)**
**Gender**	
Male	106 (22.4%)
Female	368 (77.6%)
Total	474, (100%)
**Country**	
South Africa	456 (96.4%)
Other	17 (3.6%)
Total	474, (100%)
**Occupation**	
Audiologist, speech therapist	28 (5.9%)
Clinical Associate	5 (1.1%)
Dentistry and Oral hygiene	17 (3.6%)
Homoeopath	5 (1.1%)
Medical Practitioner	76 (16.1%)
Nursing	27 (5.7%)
Occupational Therapist	62 (13.2%)
Optometrist	4 (0.8%)
Paramedic	30 (6.4%)
Pharmacist	10 (2.1%)
Physio, Chiro, Dietician, Biokineticist, Podiatry	66 (14.0%)
Radiography, sonography, radiotherapist	25 (5.3%)
Registered counsellor, psychologist, social worker	66 (14.0%)
Other	50 (10.6%)
Total	474, (100%)
**Function**	
Administrative/Managerial	61 (12.9%)
Emergency/Critical care	61 (12.9%)
Inpatient wards	88 (18.5%)
Outpatients/Primary care	264 (55.7%)
Total	474, (100%)
**Area of Work**	
Urban	306 (64.5%)
Peri-urban	80 (16.9%)
Rural	88 (18.6%)
Total	474, (100%)
**Sector**	
Public Sector	242 (51.1%)
Private Sector	232 (48.9%)
Total	474, (100%)

### Level 1: Reaction to Training

The HCWs who participated in the course reported high levels of satisfaction with their experience, providing an overall average satisfaction rating of 4.4 out of 5 (on a Likert scale from 1: very poor to 5: excellent) for the e-learning course.

### Level 2: Training Outcomes

**FIGURE 2:**
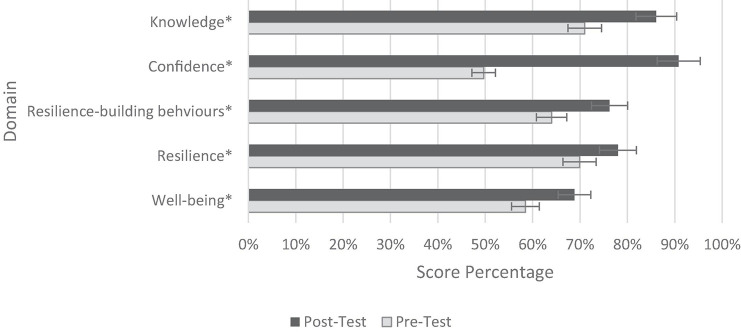
Mean pre- and post-training results. *p ≤ 0.05.

In the pre-training questionnaire, most participants were found to have high levels of knowledge (71%) and moderate levels of confidence (50%) in the learning outcomes. The post-training assessment provided evidence of statistically significant increases in the knowledge (mean increase: 1.52 points, *P*-value: 0.00) and confidence (mean increase: 4.94 points, *P*-value: 0.00) of HCWs (Figure 2).

### Level 3: Behaviour Change and Health Outcomes

Statistical analyses of the mean pre- and post-training scores revealed significant increases in self-reported resilience-building behaviours (mean increase: 3.06 points; *P*-value: 0.00), resilience (mean increase: 3.31 points; *P*-value: 0.00), and wellbeing (mean increase: 2.58; *P*-value: 0.00) of HCWs (Figure 2).

Increases in changes of behaviour and resilience scores had a significant, positive effect on change in well-being, when adjusting for the other course domains. Further analysis revealed that changes in specific behaviours influenced well-being scores (Table 2). Increasing the use of coping mechanisms for stress was associated with a 0.48-point increase in the change in well-being scores (95% CI: 0.10, 0.86) while increasing the use of self-care behaviours was associated with a 0.46-point increase change in well-being scores when adjusting for other significant factors (95% CI: 0.11, 0.80). Increasing changes in resilience scores had a positive effect on well-being scores, when adjusting for significant covariates (β = 0.20; 95% CI: 0.11, 0.29). No sociodemographic factors were statistically associated with changes in well-being score when adjusting for other covariates.

**TABLE 2:** Changes in specific behaviours and socio-demographics predicting well-being or resilience (crude and multivariable analysis).

**Variable**		**Crude analysis**		**Multivariable analysis**	
		**β** **(95% CI)**	***P*-value**	**β** **(95% CI)**	***P*-value**
Well-being^*^					
Coping mechanisms for stress		0.95 (0.64, 1.27)	**0.00**	0.48 (0.10, 0.86)	**0.01**
Mindfulness activities		0.82 (0.51, 1.13)	**0.00**	0.39 (–0.02, 0.79)	0.06
Self-care behaviours		0.66 (0.37, 0.94)	**0.00**	0.46 (0.11, 0.80)	0.01
Connecting with social support network		0.44 (0.18, 0.69)	**0.00**	0.04 (–0.26, 0.34)	0.80
Discussing impact of COVID on mental health		0.27 (–0.002, 0.58)	0.052		
Female		–0.78 (–1.65, 0.10)	0.08		
Private Sector		–0.24 (–0.94, 0.47)	0.51		
Area of work	Peri-Urban	0.63 (–0.30, 1.57)	0.18		
	Rural	0.30 (–0.64, 1.24)	0.53		
Function of work	Emergency/critical care	–0.83 (–2.24, 0.58)	0.25		
	Inpatient	–0.25 (–1.54, 1.05)	0.71		
	Outpatient	0.06 (–1.06, 1.18)	0.92		
Resilience		0.27 (0.18, 0.36)	**0.00**	0.20 (0.11, 0.29)	**0.00**
Resilience^**^					
Coping mechanisms for stress		0.81 (0.39, 1.23)	**0.00**	0.48 (0.004, 0.96)	**0.05**
Mindfulness activities		0.70 (0.29, 1.11)	**0.00**	0.09 (–0.42, 0.60)	0.73
Self-care behaviours		0.79 (0.41, 1.17)	**0.00**	0.33 (0.10, 0.76)	**0.04**
Connecting with social support network		0.53 (0.18, 0.88)	**0.00**	0.14 (–0.24, 0.51)	0.13
Discussing impact of COVID on mental health		0.25 (–0.14, 0.64)	0.21		
Female		0.49 (–0.75, 1.73)	0.44		
Private Sector		–0.45 (–1.42, 0.52)	0.36		
Area of work	Peri-Urban	–0.14 (–1.36, 1.08)	0.83		
	Rural	0.14 (–1.21, 1.48)	0.84		
Function of work	Emergency/critical care	–0.67 (–2.67, 1.32)	0.51		
	Inpatient	0.21 (–1.56, 1.99)	0.81		
	Outpatient	–0.45 (–1.97, 1.07)	0.56		
Well-being		0.37 (0.25, 0.49)	**0.00**	0.28 (0.14, 0.41)	**0.00**

*Pre-training well-being scores included in models. **Pre-training resilience scores included in models. Bold values indicate statistically significant changes.

Increases in changes in behaviour and well-being scores had a significant, positive effect on change in resilience scores, when adjusting for the other course domains. Increasing the use of specific behaviours influenced resilience scores when adjusting for other factors (Table 2). Specifically, increasing the use of coping mechanisms for stress and self-care behaviours were associated with increased changes of resilience scores (coping mechanisms for stress increase: 0.48; 95% CI: 0.004, 0.96; self-care behaviours increase: 0.33; 95% CI: 0.10, 0.76). Increasing changes in well-being scores had a positive effect on changes of resilience scores, when adjusting for significant covariates (β = 0.28; 95% CI: 0.14, 0.41). No sociodemographic factors were statistically associated with changes in resilience score when adjusting for other covariates.

## Discussion

E-learning has become an accepted approach to continuing professional development (CPD) within health profession education as the nature of e-learning is suited to the rapidly changing field and HCWs’ need for flexible delivery (Regmi and Jones, 2020). There is evidence to support the value of technology-enhanced e-learning for healthcare worker CPD (Wutoh et al., 2004; Ruiz et al., 2006; Davis et al., 2007; Cook et al., 2011; Kuiler et al., 2012; Lawn et al., 2017) and previous research has shown that technology-enhanced education is associated with improved outcomes when compared to no intervention, including effects on HCW behaviour and patient care (Cook et al., 2011).

The evaluation of this course suggests that the benefit of e-learning interventions may be observed across the first three levels of Kirkpatrick’s framework including knowledge, confidence, resilience-building behaviours, well-being, and resilience. The framework used for this evaluation is similar to the evaluation model set out by Ruggeri and Brayne (2013) which assesses the following elements of e-learning in health; baseline participant characteristics, programme outcomes and benefits, appropriateness of content for e-learning, and the cost of implementing the programme.

As expected, participants’ knowledge of stress and how to manage it improved after the course (by 1.52 points), which is comparable to the 1-point pooled effect size in the meta-analysis conducted by Cook et al. (2011). The results suggest that the training intervention contributed to an improvement in participants’ knowledge and confidence in discussing concepts related to resilience and well-being, as well as their self-reported use of resilience-building behaviours. The increase of 3.06-points found in this study is higher than pooled effect sizes reported by previous studies potentially due to the self-report nature of this study (Cook et al., 2011).

Our results are comparable to the work of Raj (2016) who found that interventions focusing on health and coping skills appear to improve well-being. This study found that increased use of coping mechanisms for stress was associated with approximately the same magnitude of change in both well-being and resilience. However, the increased use of self-care behaviours was associated with a larger change in well-being than in resilience. This finding highlights the complex relationship between resilience and well-being. Several studies have shown that resilience is positively correlated with psychological well-being and vice versa, however, the directional causality of resilience and well-being has not been clearly explored (Fredrickson and Joiner, 2002; Mak et al., 2011; Souri and Hasanirad, 2011; Rodriguez-Fernandez et al., 2018; Liu et al., 2020; Wu et al., 2020). A study conducted with Chinese college students found further support for the reciprocal relationship between resilience and mental health status, indicating that resilience predicted the level of mental health and vice versa within 1 year from baseline. In the longer term, within 2 years from baseline, mental health was found to predict the level of resilience (Wu et al., 2020). Further research needs to be conducted on the directional relationship between resilience and subjective well-being. The second phase of this study will re-assess the sample on the same domains 3 months after completion and may provide further insight into this relationship.

We observed an association between improvements in both well-being and resilience outcomes after completing the course and self-reported use of resilience-building behaviours. In our study, two specific behaviours were associated with larger changes in well-being and resilience scores: the increased use of coping mechanisms and self-care behaviours (e.g., getting adequate sleep, eating healthily, and engaging in physical activity). This is in line with previous research that suggests engaging in mindfulness and relaxation practices (Yung et al., 2004; McDonald et al., 2012; Foureur et al., 2013; Rogers, 2016b; Liu et al., 2020), getting enough sleep (Rosen et al., 2006; Kemper et al., 2015; Raj, 2016), and physical activity (Cameron and Brownie, 2010; Hart et al., 2014; Bergouignan et al., 2016) are associated with greater well-being and resilience. Although positive social relationships are also found to be associated with greater well-being (Glass, 2009; Cameron and Brownie, 2010; Hart et al., 2014; Raj, 2016; Powell, 2018; Liu et al., 2020), and this was communicated within the e-learning course, in our evaluation, connecting with social support networks did not significantly influence well-being or resilience. We posit that this may be due to the intervention taking place during the COVID-19 pandemic, under restricted social contact, limiting the ability of course attendees to “action” upon this behaviour. Behaviours related to diet, sleep and exercise may have been more within the control of participants during this time, thus more likely to be engaged with.

In our study no sociodemographic factors were statistically associated with changes in well-being or resilience scores when adjusting for other covariates. This finding suggests that HCWs from different disciplines, levels, and sectors (private vs. public) can benefit from training interventions such as this. However, research has shown that resilience is a product of complex interactions between individuals and socio ecological determinants (Liu et al., 2020). Conditions that facilitate resilience vary based on circumstances or stressors, access to available resources and socio-cultural differences (Ungar, 2017). The authors encourage future researchers to measure these for more in-depth analyses as mediators or moderators of the associations we found, alongside the inclusion of a wait-list control group.

Overall, these findings may be of particular importance to health systems and organisations interested in designing e-learning programmes to improve HCW well-being and resilience as protective factors against burnout, particularly during crises care. However, future research should focus on all level four of Kirkpatrick’s framework and rigourous evaluation methods so that the effectiveness of e-learning interventions on patient outcomes and health systems can be better understood.

## Limitations

As the primary objective of this e-learning course was to meet the immediate needs of the community, a wait-control list for research purposes was not deemed appropriate. Therefore, several limitations of the study that should be considered when interpreting the results. Convenience sampling was used as participants were included based on their willingness to participate, and there was no control group. Approximately half of the participants that completed pre-course questionnaire also completed the post-course questionnaire. This selection bias may positively skew the results as those who decided to complete the post-questionnaire may have found more benefit from the course. The results rely on self-reported data from the participants which may be susceptible to social desirability and acquiescent effects (Kreitchmann et al., 2019). However, the use of validated screening tools (CD-RISC and WHO-5) should minimise this. As the HCWs were asked to recall their behaviours and emotions over a period of 1 month, recall bias may have occurred. We recommend that future studies make use of validated tools, or objective measures where possible, to measure behaviour. The collection of post-course data was collected immediately after the course was completed; larger effects may have been seen if the participants had had more time to work on the new behaviours learnt. The second phase of the programme implementation includes another course and a follow-up survey for those that have completed the first course to assess the same domains 1–3 months after the first course. This may facilitate exploration of maintenance and comparisons between groups that have completed none, one or all of the courses.

## Conclusion

Our findings suggest that e-learning courses may improve more than competency-based outcomes alone. To fully assess the impact of an e-learning course, community programme implementors, designers, and funders, should be encouraged to build and support robust evaluation strategies that not only measure whether an improvement in knowledge and skill is achieved but also whether that knowledge and skill is applied and actioned. Finally, short- and longer-term evaluation strategies should also be considered to explore potential sustained effects of e-learning courses.

## Data Availability Statement

The datasets generated and/or analysed during the current study are not publicly available as individual privacy could be compromised but are available from the corresponding author on reasonable request.

## Ethics Statement

The study was reviewed and approved by the FPD Research Ethics Committee (certificate number: 19/2020). The FPD REC is registered with the National Health Research Ethics Council for consideration of research projects that involve minimal risk to human participants (Registration No. REC-03711-033-RA). The patients/participants provided their written informed consent to participate in this study.

## Author Contributions

FK designed the study, managed data collection, and drafted the manuscript. DB conducted the statistical analysis. MU designed the course. SM and CB provided supervisory support. All authors contributed to the article and approved the submitted version.

## Conflict of Interest

CB was employed by Johnson and Johnson Global Community Impact and SM was employed by Johnson and Johnson Health and Wellness Solutions. The remaining authors declare that the research was conducted in the absence of any commercial or financial relationships that could be construed as a potential conflict of interest.

## Publisher’s Note

All claims expressed in this article are solely those of the authors and do not necessarily represent those of their affiliated organizations, or those of the publisher, the editors and the reviewers. Any product that may be evaluated in this article, or claim that may be made by its manufacturer, is not guaranteed or endorsed by the publisher.
